# Three-dimensional histochemistry and imaging of human gingiva

**DOI:** 10.1038/s41598-018-19685-4

**Published:** 2018-01-26

**Authors:** Adriano Azaripour, Tonny Lagerweij, Christina Scharfbillig, Anna Elisabeth Jadczak, Britt van der Swaan, Manon Molenaar, Rens van der Waal, Karoline Kielbassa, Wikky Tigchelaar, Daisy I. Picavet, Ard Jonker, Esther M. L. Hendrikx, Vashendriya V. V. Hira, Mohammed Khurshed, Cornelis J. F. Van Noorden

**Affiliations:** 1 Department of Operative Dentistry, University Medical Center of the Johannes Gutenberg University Mainz, Augustusplatz 2, Mainz, 55131 Germany; 2 Department of Medical Biology, Academic Medical Center, University of Amsterdam, Meibergdreef 15, 1105 AZ Amsterdam, The Netherlands; 3 Department of Neurosurgery, Neuro-oncology Research Group, VU University Medical Center, Cancer Center Amsterdam, Room 3.36, De Boelelaan 1117, 1081 HV Amsterdam, The Netherlands; 4 Molecular Cell Biology and Immunology, VU University Medical Center, De Boelelaan 1117, 1081 HV Amsterdam, The Netherlands

## Abstract

In the present study, 3D histochemistry and imaging methodology is described for human gingiva to analyze its vascular network. Fifteen human gingiva samples without signs of inflammation were cleared using a mixture of 2-parts benzyl benzoate and 1-part benzyl alcohol (BABB), after being immunofluorescently stained for CD31, marker of endothelial cells to visualize blood vessels in combination with fluorescent DNA dyes. Samples were imaged in 3D with the use of confocal microscopy and light-sheet microscopy and image processing. BABB clearing caused limited tissue shrinkage 13 ± 7% as surface area and 24 ± 1% as volume. Fluorescence remained intact in BABB-cleared gingiva samples and light-sheet microscopy was an excellent tool to image gingivae whereas confocal microscopy was not. Histochemistry on cryostat sections of gingiva samples after 3D imaging validated structures visualized in 3D. Three-dimensional images showed the vascular network in the stroma of gingiva with one capillary loop in each stromal papilla invading into the epithelium. The capillary loops were tortuous with structural irregularities that were not apparent in 2D images. It is concluded that 3D histochemistry and imaging methodology described here is a promising novel approach to study structural aspects of human gingiva in health and disease.

## Introduction

Traditional 2D histological assessment of tissues limits proper insights in 3D tissue structure^1^. Imaging of serial sections of a tissue and subsequent 3D reconstruction of the tissue based on those images have been performed successfully, but its application has remained limited because it is a time-consuming and error-prone procedure^2,3^. Particularly, large and irregular structures such as neuronal or vascular networks are difficult to reconstruct in this way^1,3–5^. Three-dimensional imaging of tissues is hampered by the fact that tissues are opaque which limits imaging to a depth of 500–1000 µm at best^6,7^. Opaqueness of tissues is mainly caused by differences in refractive index (RI) between cell membranes and their aqueous environment^5–8^ or extracellular matrix (ECM) and their environment^9^. Therefore, a tissue-clearing step has to be included for 3D imaging of tissues.

In recent years, novel methods to clear tissues have been reported (for review, see Azaripour *et al*.^9^; Lee *et al*.^4^) and have been developed either for the removal of lipid bilayers of cell membranes^3,5,10–15^ or by impregnation of the tissue by a solvent with high RI^15–18^ to eliminate differences in RI. Most of the lipid-removal methods are focused on the central nervous system and embryos. Opacity of these tissues is mainly caused by lipid bilayers of cell membranes and not by ECM, because these tissues contain relatively small amounts of connective tissue. Clearing of ECM-rich tissues such as skin or gingiva needs an alternative approach by equalizing RIs of the different tissue compartments that are imaged^9,17^.

The German anatomist Werner Spalteholz was the first scientist who investigated clearing of tissues^19^. For the 3D study of anastomoses between coronary arteries in the heart, he developed a solution with a high RI for clearing of ECM-rich tissues. The solution consisted of methyl salicylate, benzyl benzoate (BB) and wintergreen oil. The tissue was completely impregnated with this solution. In that way, the entire tissue had obtained the same RI.

The aim of the present study was to develop methodology for 3D imaging of the vasculature in human gingiva in order to enable future studies on the effect of diabetes and smoking on the gingiva vasculature. The anatomy of the human gingival vasculature is thus far poorly described. Furthermore, investigations have usually been performed on gingiva of animals. The present study describes our investigations to optimize the methodology to stain, clear and image human gingiva using fluorescent markers, the benzyl alcohol (BA)- and BB-containing clearing solution BABB and light-sheet microscopy.

## Material and Methods

Experiments were performed on 15 samples of human gingiva that were acquired as waste patient material during periodontal surgery or tooth extraction at the Department of Operative Dentistry and Periodontology of the University of Mainz, Germany. Written informed consent was obtained from all participants and all procedures were performed in accordance with the principles outlined in the Declaration of Helsinki. All samples but one did not show signs of inflammation such as swelling, redness or bleeding as determined by one of us (AA). Histology confirmed the clinical findings as was checked by one of us (CJFVN). The inflamed sample was excluded from the study. Patients were healthy and between 40 and 60 years old. Patients were informed about the aim of the study and signed an informed consent document. After removal, samples were stored in freshly-prepared 4% paraformaldehyde (PFA; Merck, Darmstadt, Germany) in phosphate-buffered saline (PBS; pH 7.2; Gibco Life Technologies, Carlsbad, CA, USA). All experiments were performed in the Department of Medical Biology of the Academic Medical Centre (AMC) at the University of Amsterdam, The Netherlands. The entire protocol for tissue preparation, staining and clearing is shown in Suppl. 1 and is similar to the recently published protocols of Smyrek and Stelzer (2017)^20^. All incubations were performed under constant gentle shaking conditions.

### Preparation of gingiva samples

Samples were removed from the PFA solution and were washed twice in PBS. Then, samples were dehydrated in an ascending series of methanol in PBS (50%, 80%, 100%). Bleaching of the gingiva samples to reduce autofluorescence^18^ was performed at 4 °C overnight in a solution containing 1 part 30% H_2_O_2_ (Merck), 1 part DMSO (Merck), and 4 parts ice-cold methanol. Bleaching solution was replaced 3 times by 100% methanol and then twice by 20% DMSO in methanol (Merck). Finally, gingiva samples were rehydrated in a descending methanol series in PBS (80%, 50%, 0%) and were stored in PBS at room temp containing 0.1% Triton X-100 (Sigma-Aldrich, St. Louis, MO, USA). Immediately before staining, gingiva samples were blocked for nonspecific antibody binding in a solution of 5% bovine serum albumin (Sigma) in PBS at room temp.

### Staining of gingiva

Gingiva samples were incubated in a cocktail containing mouse anti-human CD31 antibodies (clone EN4; dilution 1:100; Monosan, Sanbio, Uden) as general human endothelial cell marker^21^, in PBS containing 0.2% Tween-20 (Sigma-Aldrich). YOYO-3 (Life Technologies, Carlsbad, CA, USA) in a 1:5000 dilution or 0.6 mg/ml (Hoechst 33342 Life Technologies) were used for nuclear staining. Samples were incubated at room temp for 5 days.

Before labeling with secondary antibodies, gingiva samples were washed 3 times 30 min in PBS at room temp. Then, samples were incubated in a cocktail of anti-mouse antibodies conjugated with Alexa Fluor® 568 (Life Technologies; dilution 1:100) and anti-rabbit antibodies conjugated with Alexa Fluor® 647 (Life Technologies; dilution 1:100) in PBS containing 0.2% Tween-20 for 2 days at room temp.

### Clearing of gingiva

**Figure 1:**
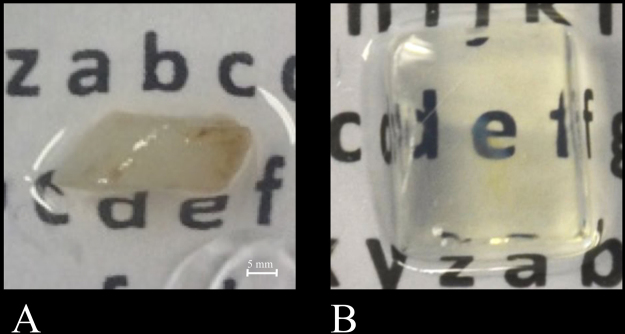
Gingiva sample before (**A**) and after clearing using BABB (**B**). After clearing, the gingiva is barely visible due to its transparency. Bar = 5 mm.

Gingiva samples were washed after staining using PBS containing 0.2% Tween-20 for 4 times at room temp. Some gingiva samples were then embedded in 2% NuSieve GTG low-melting temp agarose (Lonza, Basel, Switzerland) to facilitate handling in the microscope (Fig. 1). Samples were treated with a series of methanol in distilled water starting at 50% methanol, then followed by 70%, 80%, 96% and 3 times 100% methanol. Next, a mixture of 50% methanol and 50% BABB (BA and BB both from Sigma-Aldrich) was used for tissue storage overnight at room temp. The following morning, the methanol/BABB mixture was replaced by 100% BABB, the final clearing solution, and the samples were stored in 100% BABB at room temp.

### Imaging of gingiva

Gingiva samples were imaged using confocal microscopy (SP8-X SMD, Leica, Mannheim, Germany; 1024 × 1024 pixels; magnification: 40×) or light-sheet microscopy (LaVision BioTec, Bielefeld, Germany) with MVX10 zoom and 2× objective (Olympus, Tokyo, Japan). As dibenzyl ether (DBE, Sigma-Aldrich) is generally used as imaging medium for light-sheet microscopy and is less toxic than BABB and the RIs of BABB and DBE are similar^9^, BABB was replaced with DBE as imaging medium for some gingiva samples.

#### Confocal microscopy

Alexa Fluor ® 568 was excited at 579 nm and emission was detected at 602/30 nm, Alexa Fluor® 647 was excited at 633 nm and emission was detected at 670/30 nm, DAPI was excited at 405 nm and emission was detected at 463/50 nm.

#### Light-sheet microscopy

Alexa Fluor® 568 was excited at 545/30 nm and emission was detected at 595/40 nm. YOYO-3 was excited at 595/40 nm and emission was detected at 650/45 nm.

Three-dimensional images were taken in approx. 15 min when using light-sheet microscopy whereas imaging was performed overnight when using confocal microscopy.

### Image processing

To process the data sets, Imaris x64 7.4.2 software (Bitplane, Belfast, UK) and Fiji (ImageJ) were used^2^.

### Validation of 3D images

To validate the 3D images of the gingivae, the samples were rehydrated as follows: Incubation in 50% BABB and 50% methanol was followed by 3 times incubation in 100% methanol and a descending methanol series in distilled water (96%, 80%, 70%, 50%, 0%). Gingivae were then frozen in liquid nitrogen in Eppendorf vials. Cryostat sections were prepared using a motor-driven cryostat using a slow but constant speed (thickness, 8 µm). Cryostat sections were used for confocal imaging of fluorescence and light microscopy after hematoxylin, periodic acid Schiff (PAS) and Giemsa staining. Furthermore, PFA-fixed non-cleared samples were frozen and serial cryostat sections were prepared in a similar way. The serial sections were stained using hematoxylin. Samples were imaged using light microscopy. In the obtained images, the ascending/descending limbs of the capillary loops were visually identified and highlighted with the use of imaging software (Adobe Photoshop).

### Tissue shrinkage measurements

Gingiva samples were imaged before the start of the experiments and after preparation, staining and clearing of the samples. The images were transformed using the FIJI landmark correspondence plugin^2,3^ and a rectangle was used for reference. The outlines of the gingiva samples were drawn, and the surfaces of gingiva samples were measured. Surfaces of gingiva samples before and after preparation, staining and clearing were compared. Mean and standard deviation were calculated using Microsoft Excel (Microsoft Excel, Redmond, WA, USA). Alternatively, volumes of 2 gingiva samples were measured using Imaris software.

## Results

### Preparation, staining and clearing of gingiva samples

Preparation of the gingiva samples including bleaching to reduce autofluorescence and blocking of nonspecific antibody binding using bovine serum albumin as described in the protocol (Suppl. 1) enabled specific staining of the entire gingiva samples. The gingiva samples before clearing were checked for autofluorescence by confocal imaging and no autofluorescence was detected in the samples (data not shown).

Staining of the gingiva samples with the use of primary antibodies and the DNA dye YOYO-3, for 5 days and then secondary antibodies conjugated with a fluorescent marker for 2 days appeared to be effective. Gingiva samples were always stained throughout, including the center as was checked in each gingiva sample studied with confocal microscopy or light-sheet microscopy. All incubations were performed at room temp except for the bleaching step at 4 °C. Bacterial or fungal growth was never observed despite the long incubation periods at room temp.

Clearing of the gingiva samples as described in the protocol (Suppl. 1) resulted in complete transparency whether or not the samples were embedded in agarose for easier handling of the small samples. Figure 1A shows a gingiva sample before the procedure was started and Fig. 1B shows the same sample embedded in agarose after BABB clearing. Figure 1B shows that samples in BABB were completely transparent. Clearing occurred only in the very last step of the protocol when the sample was incubated in 100% BABB. Complete clearing occurred within 30–60 min at room temp.

We have determined quantitatively tissue shrinkage of 7 gingiva samples in BABB as compared to their size before the procedure was started in 2 ways. First, surface areas of samples were measured using FIJI software, and second, volumes of 2 samples were measured using Imaris software. The mean surface area of 5 samples was reduced by 13 ± 7% in BABB, whereas the volumes of 2 samples were reduced by 23% and 25%, respectively (13.74 mm^3^ to 10.63 mm^3^ and 6.80 mm^3^ to 5.08 mm^3^).

When the gingiva samples were rehydrated for preparation of cryostat sections for validation purposes, the samples became opaque again within 30–60 min when incubated in 50% BABB and 50% methanol.

Cleared gingiva samples were stored in BABB. In some cases, BABB was replaced by DBE because DBE is less toxic than BABB. However, the gingiva samples became brittle and hard during storage in DBE and not in BABB. Therefore, storage in BABB was used in all further experiments.

The quality of cryostat sections and the morphology of cleared gingiva samples were excellent.

### Imaging of gingiva samples

In pilot experiments, confocal laser scanning microscopy and light-sheet microscopy were compared for 3D imaging of human gingiva. First, from a practical point of view light-sheet microscopy was superior because the hydrophobic imaging medium (BABB or DBE) caused damage to the confocal microscope whereas the special tissue holders of the light-sheet microscope are BABB and DBE resistant. Second, the tissue volume that can be imaged with light-sheet microscopy (1 mm^3^–1000 mm^3^) with a spatial resolution of 1 µm appeared to be by far larger than the tissue volumes that a confocal microscope can handle. Third, imaging using light-sheet microscopy was considerably faster (15 min) than imaging using confocal microscopy (entire night). The maximum size of a tissue imaged overnight using confocal microscopy was 300 × 300 × 50 µm^3^. Fourth, out-of-focus excitation does not play a significant role when using light-sheet microscopy, whereas it does when using confocal microscopy^22^. Out-of-focus excitation is a major cause of photobleaching and photodamage^22^. Therefore, light-sheet microscopy was the imaging technique of choice for all further experiments.

### Images of gingiva samples

**Figure 2:**
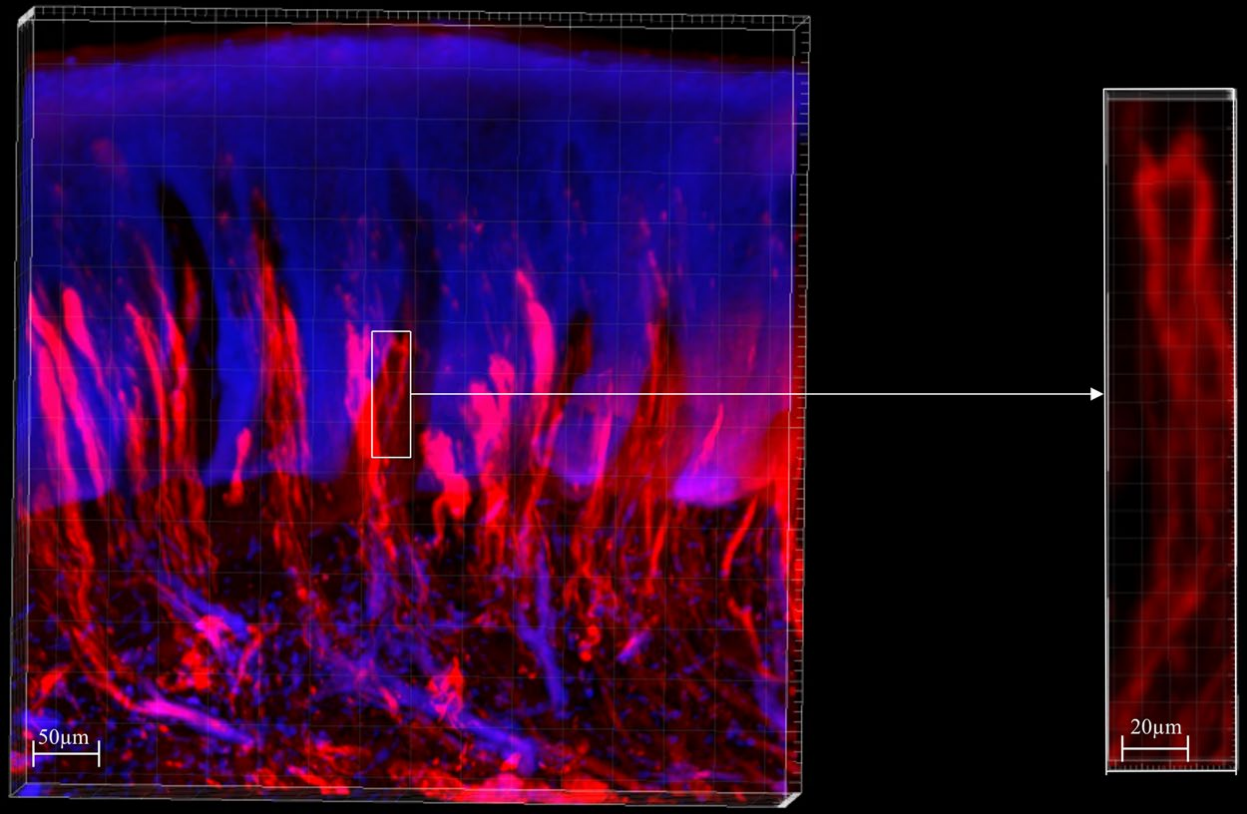
Low magnification 2D projection of a 3D image prepared with the use of light-sheet microscopy of human gingiva embedded in agarose with endothelium of blood vessels immunohistochemically stained using anti-CD31 antibodies conjugated with Alexa Fluor 568 (red) and nuclear staining using YOYO-3 (blue). Note that the colors are pseudocolors to optimize contrast. Bar = 50 µm. Detailed image bar of a capillary loop (red) in a connective tissue papilla that is invading into the epithelium of human gingiva in the box has been taken from the 3D image. Bar = 20 µm.

Figure 2 shows a 2D projection of a 3D image of human gingiva obtained with the use of light-sheet microscopy. The Suppl. Movie shows the image in 3D. The image shows stratified epithelium invaded by papillae of stroma. Each papilla contained one CD31-positive redly-stained capillary loop as is shown in the inserted higher magnification. Remarkable phenomena of the capillary loops in the papillae of all tested human gingivae was the tortuosity of the vessels and the large amount of morphological irregularities.

**Figure 3:**
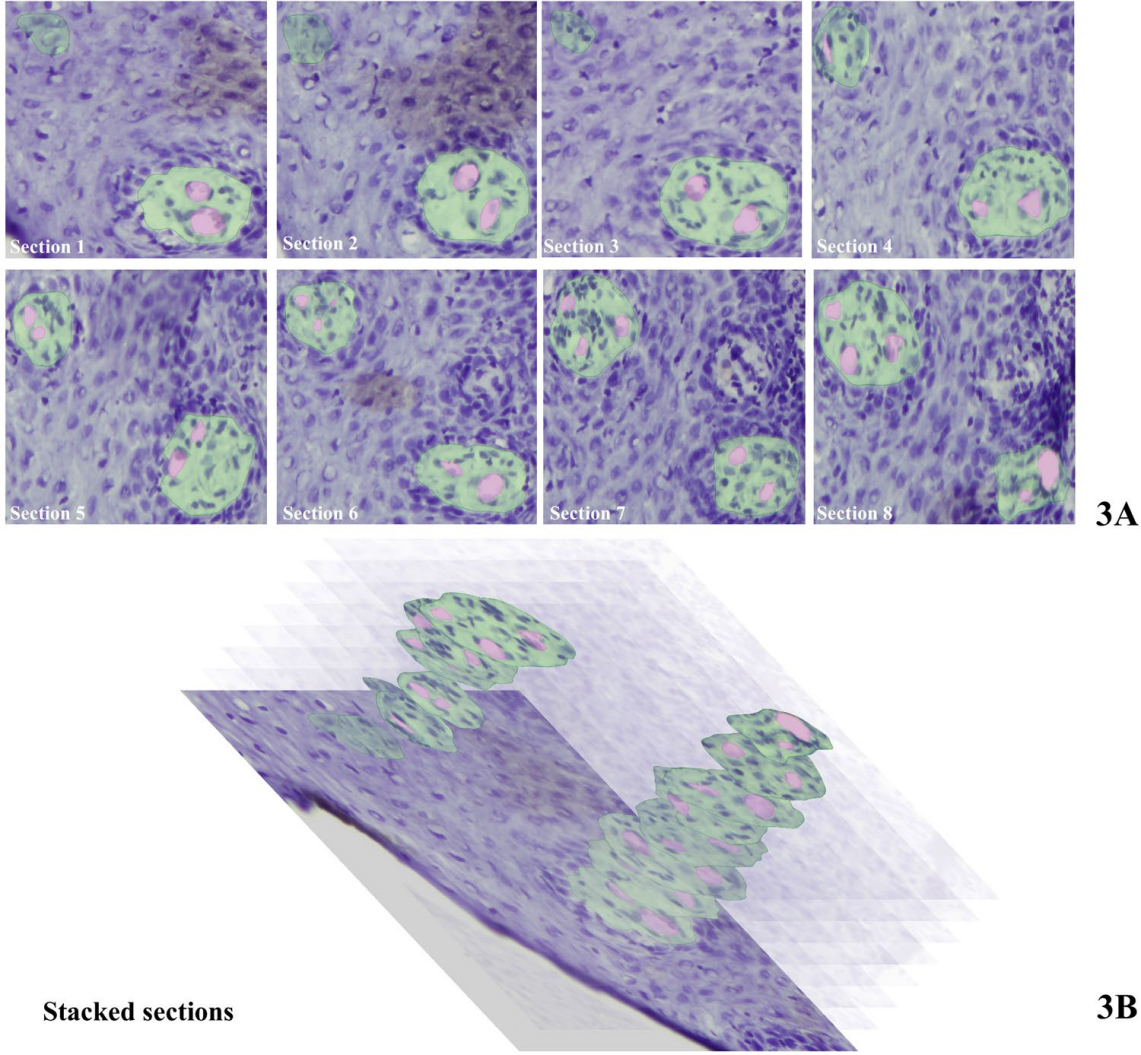
(**A**) Serial cryostat sections (8 μm thick) after staining with hematoxylin of gingiva epithelium (blue) invaded by stromal papillae (green) containing ascending and descending limbs of a capillary loop (pink) before the BABB clearing procedure. (**B**) Stacks of selections of stromal papillae prepared on the basis of the images in A. Epithelium is shown as in section 1. One papilla is sectioned from the top towards the bottom (upper papilla) and the other is sectioned halfway to the bottom. Bar = 20 μm.

**Figure 4:**
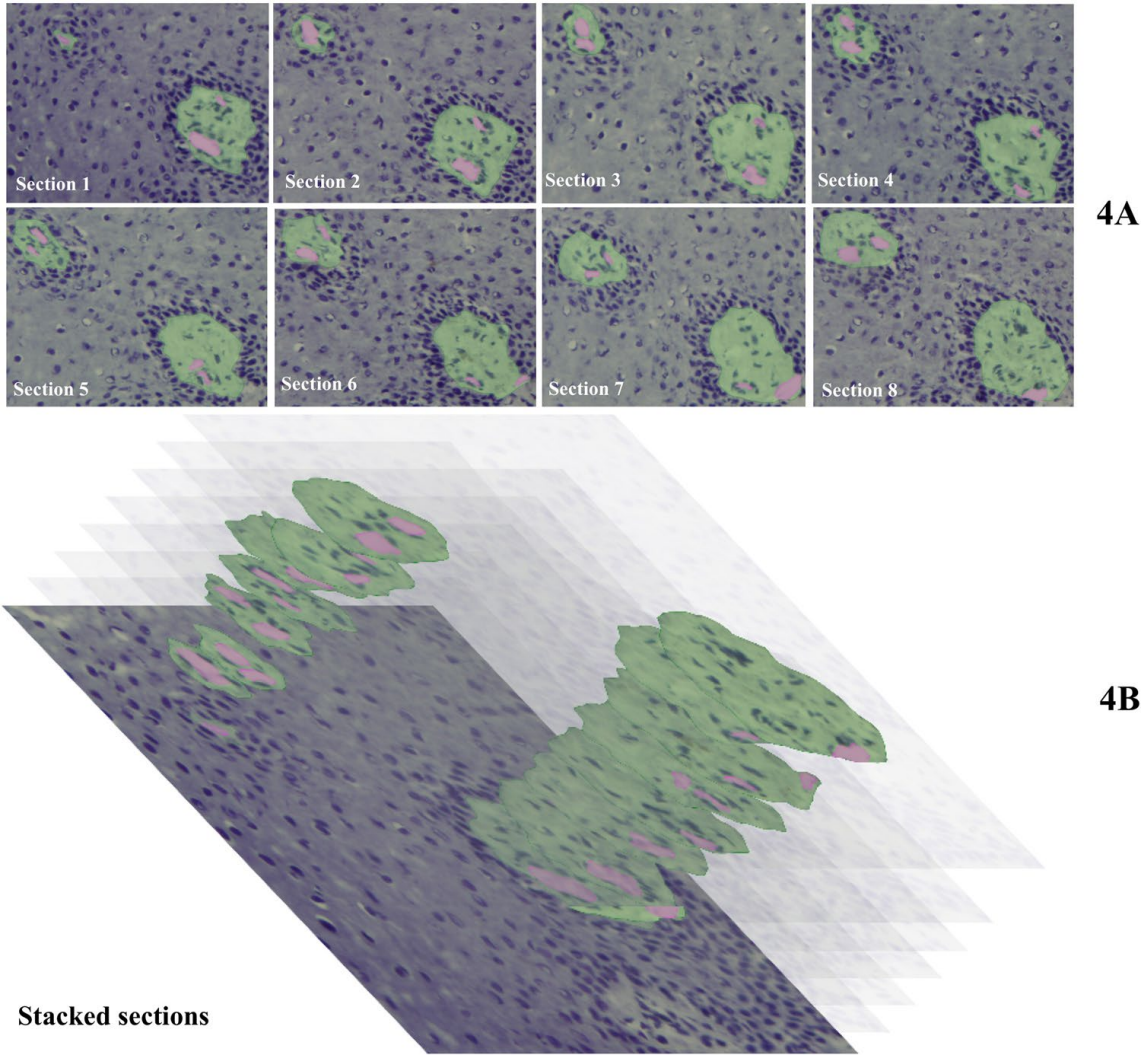
(**A**) Serial cryostat sections (8 μm thick) after staining with hematoxylin of gingiva epithelium (blue) invaded by stromal papillae (green) containing ascending and descending limbs of a capillary loop (pink) after the BABB clearing procedure. (**B**) Stacks of selections stromal papillae prepared on the basis of the images in A. Epithelium is shown as in section 1. One papilla is sectioned from the top towards the bottom (upper papilla) and the other is sectioned halfway to the bottom. Bar = 20 μm.

Figures 3 and 4 show images of 8 serial sections of gingiva epithelium each containing two transversely sectioned papillae. The capillaries in the papillae were identified on the basis of staining and morphological characteristics and highlighted with the use of imaging software (Adobe Photoshop). Figure 3 shows sections of a gingiva samples before clearing and Fig. 4 of a gingiva sample after clearing. When comparing Figs 3 and 4, it can be concluded that the BABB clearing procedure did not alter the morphology of epithelium, stromal papillae and the capillary loops in the stromal papillae. Therefore, the tortuosity and structural irregularities of the capillary loop are biologically significant and were not introduced by BABB clearing and/or tissue shrinkage due to clearing.

**Figure 5:**
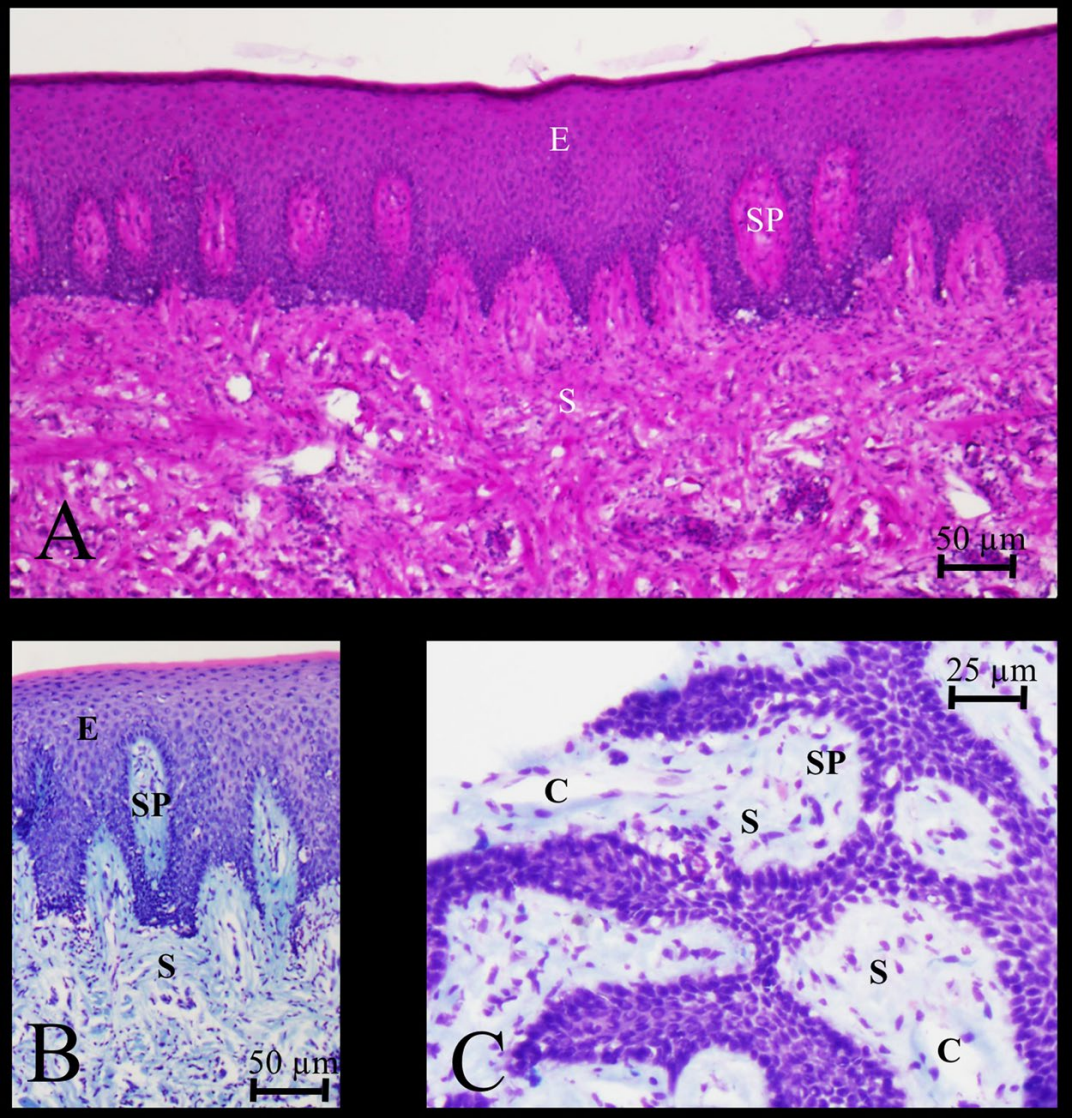
Light microscopical images of cryostat sections of the gingiva sample shown in Fig. 4. Staining of epithelium (E), stroma (S) and stromal papillae (SP) containing capillaries (C). PAS (**A**) and Giemsa (**B**,**C**) staining. Bars = 50 µm (**A**,**B**) and 25 µm (**C**).

Figure 5 shows PAS-stained and Giemsa-stained epithelium and stromal papillae invading the epithelium on top of the stroma. In the papillae capillaries are present. However, the tortuosity and irregular shape of the capillary loops remain hidden in single 2D images.

## Discussion

In the present study, 3D images of capillaries in stromal papillae invading stratified epithelium of human gingiva are shown for the first time. Clearing using BABB and imaging using light-sheet microscopy enabled 3D histochemistry and imaging of gingiva. Quantitative evaluation of capillaries and determination of changes in diameter of the blood vessels are possible using 3D histochemistry in combination with image analysis^2,3,23,24^.

Three-dimensional fluorescence images of capillary loops in stromal papillae invading into the stratified epithelium of human gingiva are a valuable tool to study gingiva in health and disease. The tortuosity and irregular shape of the capillary loops that were revealed in 3D imaging (Fig. 2) were not apparent in single 2D images (Fig. 5) but were recognized in images of serial sections (Figs. 3 and 4). This indicates the relevance of 3D histochemistry and imaging of healthy and pathological human gingiva samples. In a recent 3D visualization study of microvessels in mouse brain, a similar tortuosity and irregular shape of the microvessels was shown^24^. Likewise, it was concluded that these properties of the vascular network are missed in 2D imaging^24^.

Orban (1948) emphasized the importance of histological investigations of gingival structures as basis of comprehensive clinical interpretation and understanding of its disorders^25^. However, most studies of gingival blood vessels have been performed in animal models after perfusion with India Ink, such as the study of vascular changes during the development of gingivitis in dogs^26^. Differences in topography of the vascular system around teeth and implants have been studied in animals as well^27^. Richly-vascularized supracrestal connective tissue was found around teeth whereas supracrestal connective tissue around implants virtually lacked vascularization. Scanning electron microscopy enabled detailed imaging of the vascular network in gingiva and periodontal ligament in animal models^28^. Effects of poor oral hygiene on gingiva have been imaged *in vivo* using confocal microscopy in humans^29^. Animal studies are still necessary to investigate pathological processes in tissues when these cannot be studied in humans. However, animal studies for clinical investigations have to be limited as much as possible^30^. First, human tissues and tissues of other mammals such as mice, rats or dogs are different in many biologically-relevant aspects and evaluation and interpretation of animal studies in the light of human pathology often goes wrong. Second, restriction of the numbers of animals used in scientific research is a major moral and political issue and therefore, alternatives for animal experiments should be preferred.

The present 3D histochemistry and imaging technology promises to be a valuable tool to acquire insights in human gingival morphology and vascular structures using patient waste material. It is an easy and cheap procedure and except for light-sheet microscopy, no specialized or expensive equipment is needed. Moreover, light-sheet microscopy is becoming more and more routinely-used equipment in histology^31^. The preparation of tissues for 3D histochemistry and imaging is relatively fast with a clearing procedure of 1–4 h and immunohistochemical staining for a week, whereas fluorescence signals are well-preserved, and their quenching is limited^9,17^. An unwanted effect of BABB clearing is tissue shrinkage. Twenty percent shrinkage by BABB clearing has been reported^7,32,33^. The extent of shrinkage depends on the dehydration procedures. The faster dehydration is performed, the more shrinkage occurs, so it is advised to dehydrate slowly^16^. Our gingiva samples that have been dehydrated slowly showed shrinkage (13% reduced surface area or 24% reduced volume) which was in a similar range.

There are various clinical issues that can be addressed using 3D histochemistry and imaging^1^. It is well known that diabetic patients develop novel leaky blood vessels in retina^34^. However, only a few studies report what the effect of diabetes is on the gingival microvasculature. Keene (1969) performed a histological investigation in diabetic patients using samples of the non-inflamed palatal gingiva^35^. An increased PAS-positive staining was observed in diabetic gingival blood vessels indicating a thickening of the basal lamina of the blood vessels^36^. Besides, effects of ageing on gingival blood vessels can be explored perfectly well with 3D histochemistry and imaging. Aging effects on human gingival vascular function have been reported by Matheny *et al*. (1993) using laser Doppler flowmetry^37^. Laser Doppler flowmetry is a non-invasive method that has been used to evaluate the gingival blood flow^38–40^ However, this methodology as well as dark field imaging^41,42^ do not allow the analysis of blood vessel morphology in relation to surrounding tissues (the microenvironment) as it images the blood flow only.

Smoking is related to gingivitis, periodontitis and epithelial malignancy^43^. Smokers show a 4 times higher risk to acquire periodontitis in comparison to persons that never smoked^44^. However, studies have reported contrasting results and the mechanisms of how smoking affect periodontium are still unclear^45^.

Three-dimensional histochemistry and imaging of gingiva may well become a useful novel approach to elucidate causes of physiological and pathological changes in human gingiva in a similar manner as occurs in the retina with the use of ophthalmic imaging which can be performed *in vivo* and non-invasively because the eye is transparent^46^.

In conclusion, the procedures for preparation, fluorescence staining and BABB clearing of human gingiva samples as described in the protocol (Suppl. 1) assure that the quality and morphology of the samples are well preserved and allow 3D histochemistry and imaging of human gingiva in health and disease.

## Electronic supplementary material

Movie 1

Supplementary protocol 1
