# Supplementary material for: Three-dimensional histochemistry and imaging of human gingiva
Source: Sci Rep. 2018 Jan 26;8:1647. doi: 10.1038/s41598-018-19685-4 (PMC5785975; doi:10.1038/s41598-018-19685-4)
Supplement: Supplementary file 2 — Supplementary protocol 1 [file 41598_2018_19685_MOESM2_ESM.pdf]

Three-dimensional histochemistry and imaging of human gingiva

Adriano Azaripour[1, 2], Tonny Lagerweij[3], Christina Scharfbillig[1], Anna Elisabeth Jadczak[1], Britt van der Swaan[2], Manon Molenaar[2], Rens van der Waal[2], Karoline Kielbassa[2], Wikky Tigchelaar[2], Daisy I. Picavet[2], Ard Jonker[2], Esther M.L. Hendrikx[4], Vashendriya V.V. Hira[2], Mohammed Khurshed[2], Cornelis J.F. Van Noorden[2]

1) Department of Operative Dentistry, University Medical Center of the Johannes Gutenberg University Mainz, Augustusplatz 2, Mainz 55131, Germany
2) Department of Medical Biology, Academic Medical Center, University of Amsterdam, Meibergdreef 15, 1105 AZ, Amsterdam, The Netherlands
3) Neuro-oncology Research Group, VU University Medical Center, Cancer Center Amsterdam, Room 3.56, De Boelelaan 1117, 1081 HV Amsterdam, The Netherlands
4) Molecular Cell Biology and Immunology, VU University Medical Center, De Boelelaan 1117, 1081 HV Amsterdam, The Netherlands

Correspondence:

Dr. Adriano Azaripour, DMD, PhD, MSc

Department of Operative Dentistry, University Medical Center of the Johannes Gutenberg University Mainz, Augustusplatz 2, Mainz 55131, Germany

e-mail: adrianoasso@hotmail.com

BABB protocol

Day 1 (6 h)

**Dehydration**		
Wash in PBS	2x 60 min	R.T.
Incubate in 50% methanol in PBS	50 min	R.T.
Incubate in 80% methanol in PBS	50 min	R.T.
Incubate in 100% methanol	2x 30 min	R.T.
**Bleaching**		
Bleach in 1 part 30% H_2O_2, 1 part DMSO and 4 parts methanol at 4° C	O/N	4°C

Day 2 (7 h)

**Rehydration**		
Incubate in 100% methanol	3x 30 min	R.T.
Incubate in 20% DMSO in methanol	1x 30 min, 1x 60 min	R.T.
Incubate in 80% methanol in PBS	50 min	R.T.
Incubate in 50% methanol in PBS	50 min	R.T.
Wash in PBS	2x 45 min	R.T.
Incubate in PBS/0.2% Triton X-100	50 min	R.T.
**Blocking of nonspecific staining**		
Incubate in PBS/5% BSA	O/N	R.T.

Day 3-8 (1 h)

**Immunolabeling**		
Incubate in solution with primary antibodies in PBS/0.2% Tween-20	5 days	R.T.

Day 9 (2 h)

**Immunolabeling**		
Wash in PBS	3x 30 min	R.T.
Incubate in solution with secondary antibodies in PBS/0.2% Tween-20	2 days	R.T.

Day 10 (8 h)

Wash in PBS/0.2% Tween-20	4x 45 min	R.T.
**Dehydration**		
Incubate in 50% methanol/H_2O	50 min	R.T.
Incubate in 70% methanol/H_2O	50 min	R.T.
Incubate in 80% methanol/H_2O	45 min	R.T.
Incubate in 96% methanol/H_2O	45 min	R.T.
Incubate in 100% methanol	3x 30 min	R.T.
Incubate in 50% BABB/50% methanol	O/N	R.T.

Day 11 (1 h)

**Clearing**		
Incubate in BABB (2 parts benzylbenzoate, 1 part benzylalcohol)	Until sample is clear (30-60 min)	R.T.
